# The multifaceted role of autophagy in skin autoimmune disorders: a guardian or culprit?

**DOI:** 10.3389/fimmu.2024.1343987

**Published:** 2024-04-16

**Authors:** Yi Lin, Xiuyi Wu, Yiwen Yang, Yue Wu, Leihong Xiang, Chengfeng Zhang

**Affiliations:** Department of Dermatology, Huashan Hospital, Fudan University, Shanghai, China

**Keywords:** autophagy, skin autoimmune disorder, psoriasis, atopic dermatitis, vitiligo, systemic lupus erythematosus, alopecia areata, systemic sclerosis

## Abstract

Autophagy is a cellular process that functions to maintain intracellular homeostasis via the degradation and recycling of defective organelles or damaged proteins. This dynamic mechanism participates in various biological processes, such as the regulation of cellular differentiation, proliferation, survival, and the modulation of inflammation and immune responses. Recent evidence has demonstrated the involvement of polymorphisms in autophagy-related genes in various skin autoimmune diseases. In addition, autophagy, along with autophagy-related proteins, also contributes to homeostasis maintenance and immune regulation in the skin, which is associated with skin autoimmune disorders. This review aims to provide an overview of the multifaceted role of autophagy in skin autoimmune diseases and shed light on the potential of autophagy-targeting therapeutic strategies in dermatology.

## 1 Introduction

Autophagy also referred to as “self-eating”, is an intracellular catabolic process that involves transporting cytoplasmic components into the lysosomes for degradation and recycling (1). The primary function of autophagy is to eliminate defective organelles, untapped proteins, or intracellular pathogens, which makes it a fundamental mechanism in cellular physiology, participating in coordinated responses to stress and cell differentiation and development (2). Recent studies have revealed that autophagy is associated with several aspects of immunity, including defense against microorganisms, secretion of pro-inflammatory cytokines, and development and maintenance of lymphocytes (3, 4). Hence, autophagy is generally considered to preserve cellular homeostasis and protect against many diseases. Paradoxically, studies have suggested that autophagy also acts as a form of regulated cell death (RCD) under certain circumstances, called autophagic cell death (5), which directly contributes to the pathogenesis of numerous human diseases. Therefore, dysregulation of autophagy may cause a disturbance of cellular homeostasis and affect both innate and adaptive immunity, consequently participating in the initiation and progression of human disease.

Skin, being the largest organ of the human body, serves as the outermost protective barrier against a variety of environmental hazards, such as ultraviolet (UV) radiation, pathogen intrusion, toxic substances, and mechanical stresses (6). Additionally, the skin is recognized as an important immune organ responsible for immune surveillance and homeostasis (7). Deregulation of this function plays a significant role in the pathogenesis of many autoimmune disorders. Over the past decade, numerous studies have demonstrated that autophagy is constitutively active in various skin cell types, including keratinocytes, melanocytes, fibroblasts, and epidermal stem cells (8). Given that autophagy functions as an endogenous defense mechanism against harmful environmental factors, its activation acts as a protective mechanism by removing external stimuli, maintaining skin homeostasis, and preventing the onset and progression of skin diseases (9). Furthermore, polymorphisms in autophagy-related genes and altered expressions of autophagy-related genes and proteins have been correlated with various skin autoimmune disorders (4). Consequently, the disruption of autophagy in the skin is closely linked to disturbances in skin homeostasis and immunity, which emerge as a critical factor in the genesis and progression of skin diseases, particularly skin autoimmune disorders.

In this review, we aim to elaborate on the connection between autophagy and skin autoimmune disorders and highlight potential therapeutic approaches for these diseases employing autophagy-related mechanism.

## 2 Overview of autophagy and its molecular mechanism

Autophagy can be categorized into three types based on cargo transportation methods (1) (**Table 1**), namely, macroautophagy, microautophagy, and chaperone-mediated autophagy (CMA). Macroautophagy is marked by the generation of a double-membrane organelle termed the autophagosome, which encapsulates bulk cytoplasm and dysfunctional organelles and subsequently merges with a lysosome to degrade cargo (2). In microautophagy, lysosomes and late endosomes uptake cytoplasmic components through membrane protrusion and invagination, leading to cargo degradation within the endolysosomal lumen (10). CMA, on the other hand, operates through a chaperone-dependent degradation pathway. In the process of CMA, proteins carrying a KFERQ-like motif are recognized by the chaperone heat-shock cognate protein 70 (Hsc70), and then these proteins are transported across the lysosomal membrane via the lysosomal-associated membrane protein 2A (LAMP2A) for final degradation (11).

**Table 1:** Different classifications of autophagy.

Based on the mode of cargo transportation	Characteristics
Macroautophagy	Substrates are enclosed in cytosolic double-membrane vesicles (autophagosomes).
Microautophagy	Cytosolic components are directly incorporated into the lysosome by invagination of the lysosomal membrane.
Chaperone-mediated autophagy	Targeted proteins are transferred across the lysosomal membrane with the assistance of chaperone proteins.
Based on degradation products	Characteristics
Bulk autophagy	Indiscriminate engulfment of cytosolic components, including organelles and macromolecular complexes.
Selective autophagy	Targeting specific, often potentially harmful cargoes for degradation.

Autophagy can also be divided into nonselective and selective processes: autophagy induction that responds to nutrient deprivation operates in a nonselective (or bulk) manner, whereas starvation-independent or constitutive autophagy targets potentially harmful cargoes for degradation (12). Selective autophagy can be classified according to its targets, including mitophagy (mitochondria), ER-phagy (endoplasmic reticulum), xenophagy (intracellular pathogens), ferritinophagy (ferritin), and lipophagy (lipid droplets) (13). In selective autophagy, P62, also known as sequestosome 1 (SQSTM1), is the key adaptor protein that binds directly to LC3 on the phagophore to facilitate the targeted degradation of cargo (14). Failure to degrade these targets is associated with various diseases, and certain selective types of autophagy are also involved in other types of RCD, such as apoptosis and ferroptosis.

### 2.1 Molecular machinery of macroautophagy

Since macroautophagy is recognized as the most prevalent form of autophagy, the terms “autophagy” and “macroautophagy” are often used interchangeably (hereafter referred to as autophagy). The autophagic pathway consists of several phases, including induction, nucleation, elongation, autophagosome completion, autophagosome/lysosome fusion, and degradation, and involves multiple autophagy-related genes (*ATGs*) and their products (15). The initiation begins with the assembly of a complex made up of Unc-51-like kinase (ULK) family proteins, ATG13, ATG101, and FIP200. This complex then recruits class III phosphatidylinositol 3-kinase (PI3K) complexes, subsequently stimulating the production of phosphatidylinositol 3-phosphate (PI3P) and participating in the nucleation of phagophore (16). The next phase is elongation, which relies on two ubiquitin-like conjugation systems: the light chain 3 (LC3)–ATG8 and the ATG5–ATG12 conjugation systems (17, 18). LC3 is cleaved by the protease ATG4 to generate the isoform LC3-I. Following this, LC3-I conjugates to phosphatidylethanolamine (PE) with the assistance of ATG7 and ATG3, converting into the membrane-anchored form LC3-II (18, 19). Meanwhile, the ATG5–ATG12 conjugate system interacts with ATG16 to facilitate the conjugation of LC3-I to PE (20). Together, these two conjugation systems foster the elongation of phagophores and the formation of autophagosomes. Once formed, autophagosomes merge with an endosome or a lysosome, transforming into autolysosomes. During this fusion, the UV radiation resistance-associated tumor suppressor gene (UVRAG) protein enhances the process by interacting with the PI3K complex (21).

### 2.2 Regulation mechanisms and signaling pathways of autophagy

Autophagy is modulated by several key protein kinases, including the AMP-activated protein kinase (AMPK) and the mammalian target of rapamycin (mTOR) (22). AMPK activates autophagy directly via the phosphorylation of autophagy-related proteins, such as mTORC1 and ULK1, or indirectly by regulating the expression of downstream ATGs (23). While ULK1 activation facilitates autophagy, mTOR functions as a suppressor (23, 24). Notably, mTOR exists in two distinct complexes, namely, mTOR complex 1 (mTORC1) and mTORC2. The former hinders autophagy by phosphorylating the ULK1/Atg13/FIP200 complex, thereby inhibiting ULK1 activation (25). Recent studies have also revealed that mTORC2 participates in the regulation of autophagy through diverse downstream effectors (26). The activity of TOR kinase may be downregulated by AMPK, and the activation of the ULK1 complex is similarly attributed to the negative regulation of mTORC1 (23).

The PI3K/AKT pathway serves as the primary upstream modulator of mTORC1. When Class I PI3K is activated, it converts phosphatidylinositol-4,5-bisphosphate (PIP2) into phosphatidylinositol-3,4,5-trisphosphate (PIP3), and such transformation attracts pleckstrin homology (PH) domain proteins, such as AKT kinase (AKT) and protein kinase B (PKB), to the cellular membrane (18). Subsequently, Akt activation leads to the phosphorylation of various proteins, including mTORC1, which, in turn, inhibits the autophagic process (27). Intriguingly, different classes of PI3K exert distinct effects on autophagy. Elevated levels of the class III PI3K product, PI3P, downregulate the process (27). Beclin1, a subunit of class III PI3K complex-VPS34, is essential in stimulating PI3P synthesis and the formation of autophagosomes (22). Beclin1 can be regulated positively via ATG14L1 and the activating molecule in Beclin1-regulated autophagy 1 (AMBRA1), or negatively by anti-apoptotic proteins (15).

Accumulating evidence supports the involvement of reactive oxygen species (ROS) in mediating autophagy through a variety of signaling pathways (28, 29). Studies revealed the deactivation of Akt and mTORC1 proteins, accompanied by the activation of AMPK protein in response to oxidative stress, which subsequently triggers the initiation of autophagy machinery through the PI3K–ULK1 and Beclin1 complexes, ultimately promoting autophagy (27). Furthermore, nuclear factor erythroid 2-related factor 2 (Nrf2), a transcription factor regulating cellular antioxidant mechanisms, has been found to participate in the regulation of autophagy (27, 30). Under normal conditions, Nrf2 binds to the kelch-like ECH-associated protein 1 (Keap1), which leads to its ubiquitylation and proteasomal degradation. When ROS levels exceed the normal threshold, Nrf2 induces the expression of autophagy-related genes, including *SQSTM1/p62*, *unc-51-like kinase 1* (*ULK1*), and *autophagy protein 5* (*ATG5*), actively contributing to the enhancement of autophagy (31). On the other hand, autophagy and Nrf2 intersect in a p62-dependent manner. Autophagy dysfunction leads to p62 accumulation, which activates Nrf2 and initiates the upregulation of Nrf2-targeted genes through its competitive binding to the Nrf2-binding site of Keap1 (32). Consequently, p62-mediated Nrf2 activation and Nrf2-mediated p62 transcription may establish a positive feedback loop in the regulation of autophagy, particularly in the context of oxidative stress.

## 3 The interplay between autophagy and other cell death pathways

Generally speaking, autophagy functions primarily as a housekeeper in the maintenance of cellular homeostasis and facilitates cellular survival under various stresses. For example, in the context of oxidative stress, elevated levels of ROS can trigger protective autophagy, which effectively clears defective organelles, reduces ROS levels, and restores cellular homeostasis. Moreover, under certain conditions like nutrient depletion and hypoxia, autophagy serves to recycle essential biomolecules for cell survival and growth. Failing to do so may precipitate cell death (33).

**Figure 1:**
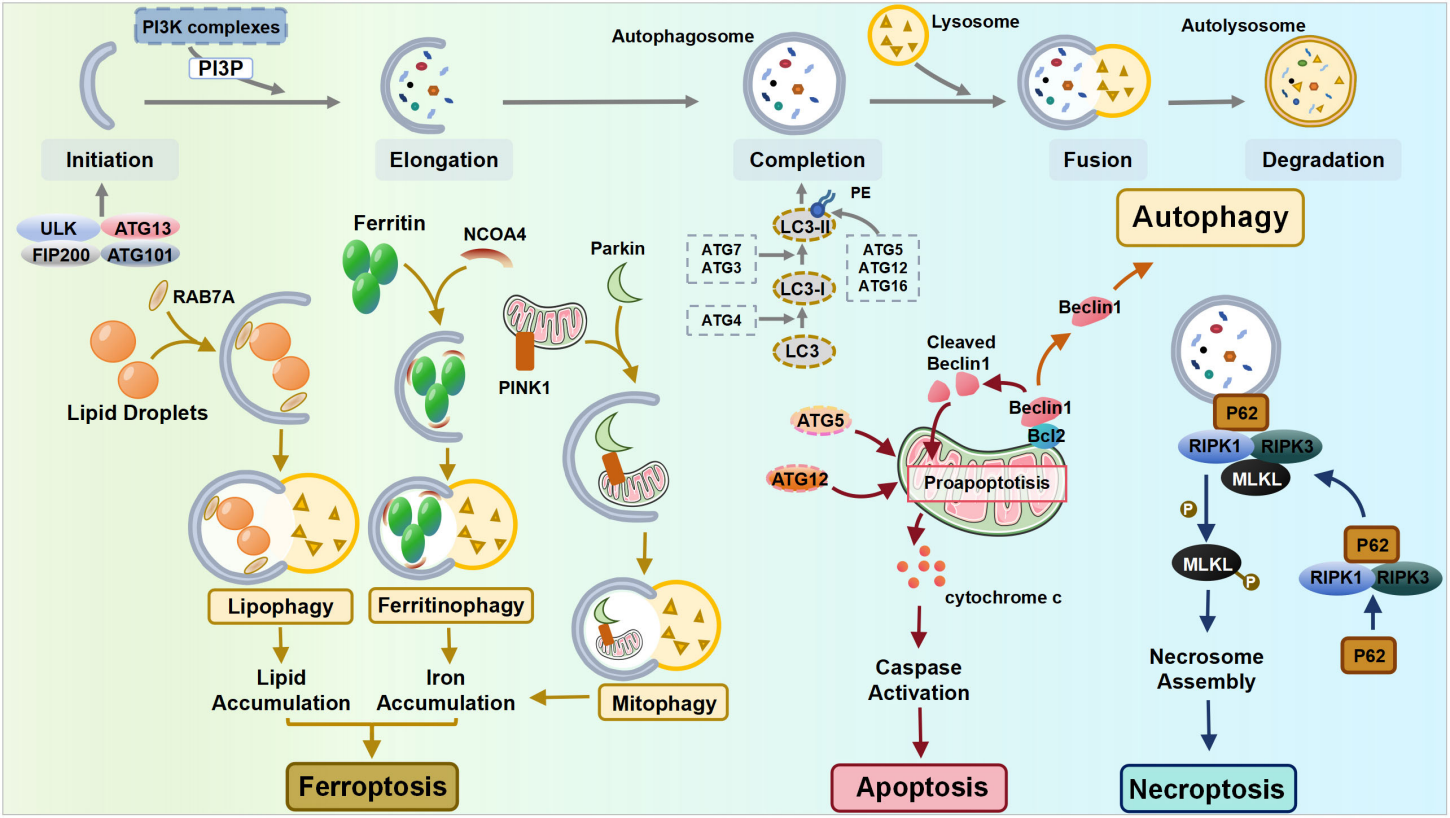
The interplay between autophagy and other cell death pathways. ULK, Unc-51-like kinase; FIP200, FAK-family Interacting Protein of 200 kDa; ATG, autophagy-related gene; NCOA4, nuclear receptor coactivator 4; PINK1, PTEN-induced putative kinase 1; RIPK, receptor-interacting protein kinase; MLKL, mixed lineage kinase domain-like pseudokinase.

However, emerging evidence suggests that autophagy also plays a decisive role in cell death processes (34) (**Figure 1**). Two distinct autophagy-related cell death modes have been identified: autophagy-dependent cell death (ADCD) and autophagy-mediated cell death (AMCD) (35). ADCD exhibits unique morphological characteristics distinct from apoptosis or necrosis and relies mechanistically on excessive autophagic machinery, such as excessive ER-phagy, excessive mitophagy, and autosis (36). ADCD has been documented in various diseases, and it has been linked to the senescence of normal human epidermal keratinocytes (NHEKs), ultimately leading to cell death (37). In contrast, in AMCD, the autophagy pathway activates various cell death modalities, including apoptosis, necrosis, and ferroptosis (34), forming a dynamic interplay with other cell death pathways.

Apoptosis is normally classified as intrinsic (mediated by Bcl-2, Bax, and Bak) or extrinsic apoptosis (mediated by membrane death receptors) based on the triggering mechanisms, while both pathways ultimately lead to the release of cytochrome c and a cascade of caspase signaling (38). Autophagy shares certain common components with apoptosis, notably through the interaction between the autophagy mediator protein Beclin1 and the anti-apoptotic protein Bcl-2. Depending on the cellular ROS level, Beclin1 can either initiate autophagy by dissociating from Bcl-2 or be cleaved by caspase when the stress signal becomes overwhelming, resulting in the suppression of autophagy. Its cleavage products subsequently lead to the release of pro-apoptotic factors from mitochondria, hastening the process of apoptosis (27, 39). Additionally, autophagy-related molecules can directly interact with apoptotic molecules, thereby promoting apoptosis. The Atg5 and Atg12 conjunction system is essential for autophagy, while recent discoveries have identified that the unconjugated forms of Atg5 and Atg12 can promote apoptosis independently (40).

Necroptosis is an inflammatory form of RCD, whose mechanisms involve receptor-interacting protein kinase 1 (RIPK1), RIPK3, and mixed lineage kinase domain-like pseudokinase (MLKL). The autophagy machinery can serve as a scaffold, allowing for more efficient activation of the necrosome by recruiting RIPK1 through p62. This recruitment leads to the phosphorylation of MLKL, ultimately leading to cell necroptosis (41).

Ferroptosis, a recently identified form of RCD, is activated mainly by the accumulation of iron and lipid peroxide production. Autophagy, particularly selective autophagy, plays a crucial role in the mechanisms of ferroptosis, including the regulation of cellular iron homeostasis, lipid metabolism, and redox homeostasis (42, 43). Upon the induction of ferroptosis, autophagy is triggered, leading to the degradation of ferritin and the ferritinophagy cargo receptor, nuclear receptor coactivator 4 (NCOA4). This process results in elevated levels of cellular labile iron and the rapid accumulation of cellular ROS, both of which are essential for the machinery of ferroptosis (44). In addition to ferritinophagy, lipophagy is also correlated with ferroptosis, as the level of lipid droplets shows a negative association with ferroptosis (42). Mediated by RAB7A (a member of the RAS oncogene family), lipophagy recruits lipid droplets via multivesicular bodies and lysosomes. The increased RAB7A-dependent lipophagy promotes lipid droplet degradation, thereby facilitating lipid peroxidation-mediated ferroptosis (42). Furthermore, glutamine metabolism, the tricarboxylic acid cycle, and the electron transport chain play pivotal roles in cysteine deprivation-induced ferroptosis, suggesting that compromised mitochondria contribute to ferroptosis (45, 46). Therefore, under the condition of cysteine deprivation, PTEN-induced putative kinase 1 (PINK1)-mediated mitophagy can specifically degrade excess or dysfunctional mitochondria within cells, serving as a negative regulator of ferroptosis (47, 48). However, as mitochondria also regulate intracellular redox homeostasis and iron metabolism, the excessive activation of mitophagy facilitates ferroptosis through the release of free Fe^2+^, which allows the Fenton reaction to proceed with unreduced ROS (49).

Collectively, autophagy has a multifaceted role in various cell death mechanisms and a more in-depth exploration of the precise regulatory mechanisms of autophagy is required to reach a better understanding of the significance of the interplay between autophagy and other cell death pathways.

## 4 The crosstalk between autophagy and autoimmunity

Autophagy and autophagy proteins have emerged as vital players in a diverse array of immune functions, including host defense against pathogens, regulation of immune cell development, and antigen processing and presentation (50, 51). Autophagy participates in the regulation of both innate and adaptive immunity.

### 4.1 Autophagy, innate immunity, and inflammation

Autophagy has been reported to modulate the innate immune system by regulating innate immune cell differentiation, phagocytosis, cytokine secretion, and antigen processing and presentation, which not only shapes the innate immune response but also affects the activation of the adaptive immune compartment (52).

Autophagy exerts diverse influences on different innate immune cell types. For example, constitutive autophagy promotes macrophage differentiation at various stages, while negatively impacting the development of neutrophils (53). Moreover, autophagy is actively involved in the process of phagocytosis by promoting the fusion of phagosomes with lysosomes, which enhances the clearance of pathogens, foreign material, or dead cells (54). Autophagy is also integral to the process of antigen presentation of both MHC class I and II molecules by antigen-presenting cells (APCs) (29). Autophagy affects the production of antigenic peptides and the expression of MHC class I molecules on the cell surface. During the MHC class II molecular antigen presentation, antigens captured by APCs are transported to the autophagosomes to further produce immunogenic peptides (29). Therefore, in dendritic cells (DCs) and macrophages, which function as professional APCs, the induction of autophagy promotes both MHC class II and MHC class I antigen presentation (50).

Another notable connection between autophagy and the innate immune response is cytokine secretion. Blocking autophagy increases interleukin (IL)-1β and IL-18 production in macrophages, thereby promoting inflammasome activation (55). Autophagy-dependent cytokine production was also observed in DCs, involving key cytokines such as IL-6, tumor necrosis factor (TNF)-α, and interferon (IFN)-γ (52), mediating inflammatory and immune responses.

### 4.2 Autophagy and adaptive immunity

In the adaptive immune response, autophagy and autophagic proteins are not only essential to antigen presentation and thymic selection, but also vital to the development, survival, and homeostasis of T and B lymphocytes. The targeted knockout of different autophagy genes in specific lymphocyte populations has demonstrated an indispensable role for autophagy proteins in the maintenance of normal numbers of B1 B cells, CD4^+^ T cells, and CD8^+^ T cells in mice (56, 57).

With regard to the development and survival of B cells, autophagy-related genes and signaling pathways are related to the transition of pro-B cells to pre-B cells and autophagy is required for the peripheral self-renewal of B1 cells (58–60). In the context of T cell development and survival, where mitochondrial content is developmentally reduced during the transition from thymocyte to peripheral T cell, the absence of autophagy could result in developmental defects of T cells, potentially related to the impaired clearance of mitochondria (61). Autophagy also plays a role in T cell differentiation. Specifically, defective autophagy has been proven to inhibit the transition from a double-negative to a double-positive stage of the T cells in the thymus (62). T cells that have undergone positive selection interact with thymic epithelial cells (TECs), consequently resulting in the elimination of autoreactive T cells (63). High levels of autophagy in TECs are instrumental in the delivery of self-antigens to MHC class II loading compartments (56, 62). Genetic disruption of Atg5 in TECs alters the selection of certain MHC class II-restricted T cell specificities and autoimmunity (64). Moreover, autophagy promotes T cells to evolve into invariant natural killer T cells and regulatory T cells (Treg) in the thymus (65). It also facilitates the differentiation of CD8^+^ T cells into cytotoxic T lymphocytes and guides T cells to differentiate into T helper cells in the periphery (53).

Furthermore, as previously mentioned, MHC class II-loading compartments can fuse with autophagosomes (66). Therefore, autophagy proteins may actively participate in various facets of antigen presentation, facilitating the delivery of endogenous antigens for MHC class II presentation to CD4^+^ T cells, in addition to enhancing the cross-presentation of antigen donor cells to CD8^+^ T cells (67).

Autophagy modulates the development, homeostasis, and functions of B and T lymphocytes. Nevertheless, the cytokines secreted by lymphocytes in turn influence the progress of autophagy. Specifically, transforming growth factor (TGF)-β, interferon (IFN)-γ, IL-1, IL-2, and IL-12 are autophagy inducers while IL-4, IL-10, and IL-13 are autophagy inhibitors (53), further delineating the intricate interplay between autophagy and adaptive immune system components.

## 5 Autophagy in skin autoimmune disorders

### 5.1 Psoriasis

Psoriasis is a chronic immune-mediated inflammatory skin disease that is clinically characterized by the presence of red, scaly plaques or patches and is often accompanied by common comorbidities such as metabolic syndrome and cardiovascular disease. Although the exact etiology of psoriasis is not yet fully understood, it is believed to be caused by a combination of genetic and environmental factors (68). Recent studies have revealed that mutations in the autophagy regulator genes *ATG16L1* and *AP1S3* are associated with psoriasis (69, 70). The connection between autophagy dysfunction and psoriasis pathogenesis was further verified by the altered expression of autophagy-related proteins in psoriatic skin, such as ATG5, ATG7, Beclin1, LC3, and p62 (71–75).

Hyperplasia and abnormal terminal differentiation of epidermal keratinocytes are major pathological characteristics of psoriasis (76). As autophagy is constitutively active in the epidermis and modulates the terminal differentiation and proliferation of keratinocytes, its impairment contributes to aberrant keratinocyte proliferation in psoriasis (77). LC3 expression was downregulated or even absent in psoriasis lesional skin epidermis, negatively correlating with the mean thickness of the epidermis, indicating the impairment or blockade of autophagy in psoriasis (74, 78, 79). Controversially, several other studies indicated the elevated expression of LC3 and other autophagy-related factors including ATG5 and ATG7, which may point to the induction of autophagy (72, 75, 80). It was speculated that the increased autophagosome formation could be a compensatory mechanism for reduced autophagic degradation activity, as evidenced by the accumulation of p62 and the decline of lysosome protease activity in patients with psoriasis, leading to parakeratosis in psoriasis (75, 78, 79). Abnormal keratinocyte proliferation and differentiation cause defects in the epidermal barrier and the microorganism defense mechanism. Meanwhile, autophagy dysregulation has also been related to inefficient bacterial clearance (6), with studies revealing the crucial role of bacteria colonization, such as *Staphylococcus aureus* and *Streptococcus danieliae*, in exacerbating psoriasis (76).

Inflammation is another hallmark of psoriasis pathogenesis and accumulating evidence has demonstrated the involvement of autophagy in modulating the immune response. Defective autophagy contributes to an upregulation of proinflammatory transcription factors (e.g., NF-κB) and the production of proinflammatory cytokines (e.g., IL-1β and IL-36) (70). The decreased level of LC3 in the psoriatic epidermis is negatively correlated with inflammatory cell infiltration (79). IL-17A, a crucial cytokine in the disease pathogenesis, has been shown to stimulate autophagosome formation in early stages; however, upon longer-term exposure to IL-17A in keratinocytes, it inhibits autophagy through the activation of the PI3K/AKT/mTOR signaling pathway, indicating a dynamic crosstalk between autophagy and inflammation in psoriasis (78). Feng et al. reported that cis-khellactone inhibited the activation of NF-κB and the infiltration of macrophages by promoting autophagy, which subsequently ameliorated imiquimod-induced psoriasis in mice (81). Daturataturin A has also been confirmed to induce autophagy, which negatively regulates inflammation in human immortalized keratinocytes (82). Collectively, these studies suggest that autophagy takes on a proactive role in psoriasis and that its dysfunction exacerbates the disease. However, AMPK was found to promote both autophagy and skin inflammation in the psoriasis mouse model via the ULK1/ATG7 signaling pathway (83). Moreover, a recent study demonstrated that MAPK pathway-activated autophagy exacerbated skin inflammation in patients with psoriasis and mouse models with psoriasis, whereas autophagy blockade alleviated inflammation. The study also identified the autophagy-based release of high mobility group box 1 (HMGB1) as a booster of psoriatic inflammation (80). In conclusion, dysregulation of autophagy may contribute to the pathogenesis of psoriasis, making autophagy a potential target for therapeutic intervention in psoriasis.

### 5.2 Atopic dermatitis

Atopic dermatitis (AD) is a relatively common inflammatory skin disorder that is characterized by impaired epidermal barrier function and an excessively activated immune system (84). Emerging evidence has demonstrated that autophagy is implicated in the pathogenesis of AD. RNA sequencing analysis has revealed increased expression of autophagy-related genes in AD patients, such as *ULK1*, *ATG4*, and *ATG16L2* (85). Decreased LC3 levels and increased p62 levels have been observed in the epithelium of both patients and AD mouse models compared to healthy controls and normal mice, reflecting an autophagic blockade in the pathogenesis of AD (86). In a different study, however, raised levels of ATG5, ATG7, LC3B, and p62 were detected in the epithelium of AD patients (75). Although the elevation of the first three proteins indicates autophagy induction, it could represent a cellular response to compensate for the decline in autophagic degradation activity by increasing autophagosome formation. Moreover, levels of functional lysosome proteases, cathepsins D and L, were significantly reduced in the epithelium of patients with AD. In general, these results supported the pathogenic role of defective autophagy in AD.

Inflammation also plays a pivotal role in the pathogenesis of AD, with multiple immune cells and pro-inflammatory cytokines jointly contributing to the inflammatory response in AD. TNF-α, which exhibits proinflammatory effects in AD, is able to induce autophagy in the early phase to re-establish cellular homeostasis, but prolonged exposure to it inhibits lysosomal activity and autophagy flux in keratinocytes (75, 87). Studies have shown that Th2 cytokines (e.g., IL-4 and IL-13), which were upregulated in AD skin lesions, counteracted autophagy induction in human keratinocytes by activating the mTOR pathway (86, 88). IL-37, on the other hand, increases AMPK levels and thereby leads to a reduction in the expression levels of mTOR, ultimately promoting autophagy and ameliorating inflammation in AD (89).

Furthermore, skin barrier disruption, another hallmark of AD, has also been associated with dysfunctional autophagy. Defective autophagy suppresses the differentiation of keratinocytes and downregulates stratum corneum barrier-related and tight junction barrier-related proteins, jeopardizing the integrity of the skin barrier (86, 90). Moreover, autophagy is also essential for the skin’s defense mechanisms against invading pathogens. Dysbiosis of the skin microbiota, especially *S. aureus*, has been found to exploit autophagy and thereby persist within keratinocytes, contributing to the pathogenesis of AD as well (91). Hence, dysfunctional autophagy significantly contributes to the pathogenesis of AD by causing damage to the epidermal barrier and perpetuating inflammation.

### 5.3 Vitiligo

Vitiligo is one of the most important pigmentary disorders, characterized by the absence or reduction of functional melanocytes in the epidermis, resulting in depigmented skin lesions. Despite the obscure etiology, proposed mechanisms so far include oxidative stress, autoimmune responses, genetic factors, and neural influences (92).

Autophagy has been associated with the biological functions of melanocytes, including melanin metabolism and the formation, maturation, and destruction of melanosomes (93). Various autophagy-related proteins, such as ATG7, ATG4, LC3, and Beclin1, are strongly correlated with the melanogenesis pathway (94, 95). Autophagy activation and increased levels of LC3 have been linked to enhanced melanin synthesis in melanocytes (96). In contrast, LC3 depletion suppressed the expression of microphthalmia-associated transcription factor (MITF) and tyrosinase, resulting in decreased melanin content, which suggests that impaired autophagy may participate in the pathogenesis of vitiligo (96). Furthermore, autophagy inducers, such as lipopolysaccharide and 30-hydroxydaidzein, stimulated melanogenesis in melanocytes and PIG3V melanocytes (a vitiligo melanocyte cell line), while autophagy inhibitors, such as 3-methyladenine or chloroquine, exerted the opposite effect (97).

Dysregulated autophagy disrupts the antioxidant defense system in melanocytes, ultimately contributing to the onset and progression of vitiligo (98). Studies have demonstrated that vitiligo melanocytes exhibited impaired autophagy and increased proneness to hydrogen peroxide-induced oxidative stress (99). In addition, specific suppression of ATG7-dependent autophagy in melanocytes failed to exhibit any discernible effect on pigment production or the formation and maturation of melanosomes. Instead, the absence of autophagy in melanocytes impeded their proliferation, disrupted Nrf2 signaling, and reduced the antioxidative defense mechanism within melanocytes (100, 101). On the other hand, the upregulation of ATG7-dependent autophagy defends melanocytes from oxidative stress-induced apoptosis (101). Collectively, ATG7-dependent autophagy is essential for maintaining normal biological processes and redox balance in melanocytes.

In a recent study employing RNA sequencing to analyze tissue samples from vitiligo patients, autophagy inhibition was observed in lesional skin, as supported by the decreased ratio of LC3-II/LC3-I and increased p62 expression in vitiligo lesions (102). Moreover, polymorphisms of the *UVRAG* genes may contribute to enhanced susceptibility to non-segmental vitiligo, connecting autophagy dysregulation with vitiligo pathogenesis (103). Intriguingly, autophagy induction has also been reported in vitiligo. Bastonini et al. demonstrated elevated expression levels of autophagic markers, including LC3-II, ATG7, ATG8, and ATG5, in non-lesional vitiligo melanocytes compared to normal melanocytes. Concurrently, p62 levels were reduced. These observations could be attributed to the mitochondrial defects and the subsequent impaired energy metabolism (104). On the other hand, inhibition of autophagy leads to an exacerbation of the deleterious metabolic effects observed in the corresponding melanocytes, further suggesting that autophagy in non-lesional vitiligo melanocytes is a responsive mechanism to metabolic surveillance (104). In comparison to active vitiligo lesions, stable lesions exhibited increased levels of autophagy, which suggests that the induction of autophagy serves as a protector to counteract the progression of the disease (105).

Considering that vitiligo is now viewed as a disease affecting the entire skin, not just melanocytes, the alteration of autophagy may extend to other skin cells including keratinocytes and fibroblasts (102, 106). Therefore, the variable role of autophagy in vitiligo reflects complex mechanisms, that ultimately lead to melanocyte loss, and is in need of further investigation.

### 5.4 Systemic lupus erythematosus

Systemic lupus erythematosus (SLE) is a chronic autoimmune disease characterized by the immune system mistakenly attacking healthy cells and tissues due to a failure to distinguish between certain self-antigens and foreign ones (107). The etiology and pathogenesis of SLE are complicated and multifactorial, involving mechanisms such as genetics, environmental elements, hormonal factors, and abnormal immune function. In SLE, an elevated level of cell apoptosis has been associated with an increase in autoantigens, which subsequently induces an excessive autoimmune response (108). Autophagy has been implicated in the degradation of pro-apoptotic proteins and clearance of apoptotic cells, thereby effectively preventing the onset of inflammation. Recently, autophagy dysfunction has gained gradual recognition as a contributing factor in SLE (109, 110). Studies focusing on genome-wide association have identified several autophagy-related genes associated with SLE susceptibility, such as *ATG5*, *ATG16L2*, *CDKN1B*, *DRAM1*, and *CLEC16A* (111). Additionally, polymorphisms of the *ATG5* gene and the *Prdm1-ATG5* intergenic region have also been connected to SLE, further implicating the involvement of autophagy in this disease (112).

The dysregulated immune response plays a critical role in the development and progression of SLE, which is characterized by abnormal B and T cell activation, cytokine secretion, and autoantibody production (113). Dysregulation of autophagy affects both innate and adaptive immune responses in SLE and contributes to the disease’s pathogenesis through multiple mechanisms. First, autophagy regulates the development, proliferation, and activation of T and B cells (114). Under normal circumstances, autoreactive B cells will be eliminated through apoptosis. Upregulated autophagy, on the other hand, enables autoimmune B cell precursors to evade the initial tolerance checkpoints and escape physiological deletion (115). In patients with SLE, defective B cell tolerance checkpoints have been observed, along with intensified autophagy in B cells, especially in naïve B cells (116). Accordingly, levels of anti-nuclear antibodies (ANA) and inflammatory cytokines were reduced in mice with B cell-specific ablation of autophagy, as well as inhibited plasma cell (PC) differentiation, suggesting that autophagy may regulate the survival of autoreactive B cells and the differentiation of PCs in SLE (117).

Autophagy also appears to participate in the processing and presentation of self-antigen-derived peptides to cognate T cells (118). Using an autophagy inhibitor, researchers observed reduced MHC class II molecules in B cells, a diminished number of PCs, and decreased IgG secretion by lupus B cells (119). Moreover, higher numbers of autophagic vacuoles were noted in T cells of lupus-prone mouse models and patients with SLE, particularly in peripheral T cells (120). This observation indicates that autophagy may regulate the survival of autoreactive T cells in individuals with lupus. In a more recent study, CD4^+^ naïve T cells extracted from patients with SLE exhibited elevated levels of constitutive autophagy compared with those from healthy controls (121). However, other studies have indicated a blockade of autophagy in SLE, rather than activation. It has been confirmed that autophagy is necessary for T cell differentiation, activation, and survival, the downregulation of which can lead to mitochondrial dysfunction, increased production of ROS, and, ultimately, cell death (122). Indeed, mTOR signaling is enhanced in SLE, which subsequently inhibits autophagy and is considered to be a central mediator to lupus pathogenesis (122). Furthermore, T cells from patients with SLE were demonstrated to be resistant to autophagy induction, and autophagy-suppressing genes (e.g., *Bcl-2* and *Akt1*) were overexpressed (121). Failure to induce autophagy in SLE could lead to an overload of damaged mitochondria and thus excessive ROS production. In support of the notion that autophagy inhibition is involved in SLE pathogenesis, Fernandez et al. found that rapamycin, an autophagy inducer, was able to alleviate disease activity and restore T cell activation in patients with SLE (123). Therefore, the function of autophagy in patients with SLE can be cell type-dependent and have distinctive roles in different stages of the same cell.

Elevated levels of IFN-α have been acknowledged as a hallmark of SLE, and the secretion of it from plasmacytoid DCs is modulated by autophagy (50, 124). As skin lesions in patients with SLE exhibit photosensitivity, UV exposure is also closely associated with the development of SLE. The regulation of apoptotic cells and DNA damage clearance through autophagy is crucial in preventing the development of SLE, as defects in this process may promote the disease (125). In addition, infections, especially Epstein–Barr virus (EBV), are another triggering factor for SLE. EBV-encoded latent membrane protein 1 has been observed to induce autophagy, facilitate the activation of B and T cells, and might lead to abnormal immune responses (126). In summary, the deregulation of autophagy could be integrated into many aspects of pathogenesis.

### 5.5 Alopecia areata

Alopecia areata (AA) is an autoimmune disease that primarily targets hair follicles (HFs), resulting in non-scarring hair loss (127). Autophagy has been proven to be involved in the modulation of hair growth. Specifically, activated autophagy encourages hair growth in dormant telogen HFs, whereas inhibiting autophagy leads to apoptosis-driven involution of HFs *ex vivo* (128, 129). In addition, autophagy promotes the differentiation of HF stem cells (130). Genome-wide association studies have identified genetic variations related to autophagy regulation, such as *STX17*, *CLEC16A*, and *BCL2L11*, as predisposing genetic loci for AA (131, 132). Furthermore, several AA patients have exhibited copy number variations (CNVs) in the genomic region spanning *ATG4B*, another key autophagy gene (133).

Notably, disruptions in autophagy have been implicated in the immunologic derangement in the pathogenesis of AA (134). Advanced-stage AA is marked by reduced autophagic activity, as evidenced by the accumulation of SQSTM1 in the affected skin and HFs of AA mouse models, in contrast to both non-lesional and healthy skin and HFs. Conversely, notably higher levels of ATG5 and LC3B in non-lesional HFs compared with healthy ones may reflect a temporary autophagic upregulation in the early stages of AA pathogenesis. Pharmacological induction of autophagy, on the other hand, restores autophagic activity and reduces the associated inflammation in AA skin, while autophagy inhibition accelerates disease onset (135, 136). Recent research identified melanocytes as the ignitor of autoimmune attacks in both vitiligo and AA and proposed that alterations in the autophagy mechanism participate in the process (134, 137). Taken together, these results reveal the involvement of disrupted autophagy in the pathogenesis of AA.

### 5.6 Systemic sclerosis

Systemic sclerosis (SSc) is an autoimmune disease characterized by skin and visceral organ fibrosis as well as vasculopathy, resulting from aberrant activation of fibroblasts, excess deposition of collagen, and abnormal fibrosis. The pathogenesis of SSc is intricate and may involve genetic and environmental factors, along with immune dysregulation (138). Malfunctional autophagy is believed to play a key role in SSc, as evidenced by the upregulation of autophagy in the fibrotic skin of SSc patients with increased expression levels of LC3, Beclin1, and ATG7, in addition to downregulated protein levels of p62 (108, 139). Similarly, activation of autophagy in fibroblasts was also observed in murine models of pulmonary or dermal fibrosis, especially in the sclerotic phase compared to the edematous phase, suggesting an association between abnormal activation of the autophagic degradation system and fibrosis in SSc (139, 140). Moreover, inhibition of the PI3K/Akt/mTOR signaling pathway reduced the production of the fibrotic cytokine connective tissue growth factor (CTGF) and collagen I in SSc fibroblasts via downregulation of HIF-1α (141). Meanwhile, the treatment of a dual inhibitor for PI3K/Akt and mTOR signaling in an SSc mouse model also attenuated dermal fibrosis, reinforcing the role of PI3K/Akt/mTOR signaling in SSc (142).

Among the cytokines implicated in SSc, transforming growth factor-β (TGF-β) is considered the main profibrotic molecule, functioning through the activation of the small mother against decapentaplegic (SMAD) signaling and the production of the extracellular matrix (ECM) (143). Zehender et al. proved that inhibition of autophagy renders human fibroblasts less sensitive to the profibrotic effects of TGF-β, thereby reducing fibroblast activation and ameliorating fibrosis (139). Specifically, TGF-β activates autophagy by repressing MYST1, which further unravels the possible mechanism of TGF-β and autophagy in the context of fibrosis. These findings suggest that regulating autophagy may serve as a potential therapeutic approach for SSc.

## 6 Conclusion

**Figure 2:**
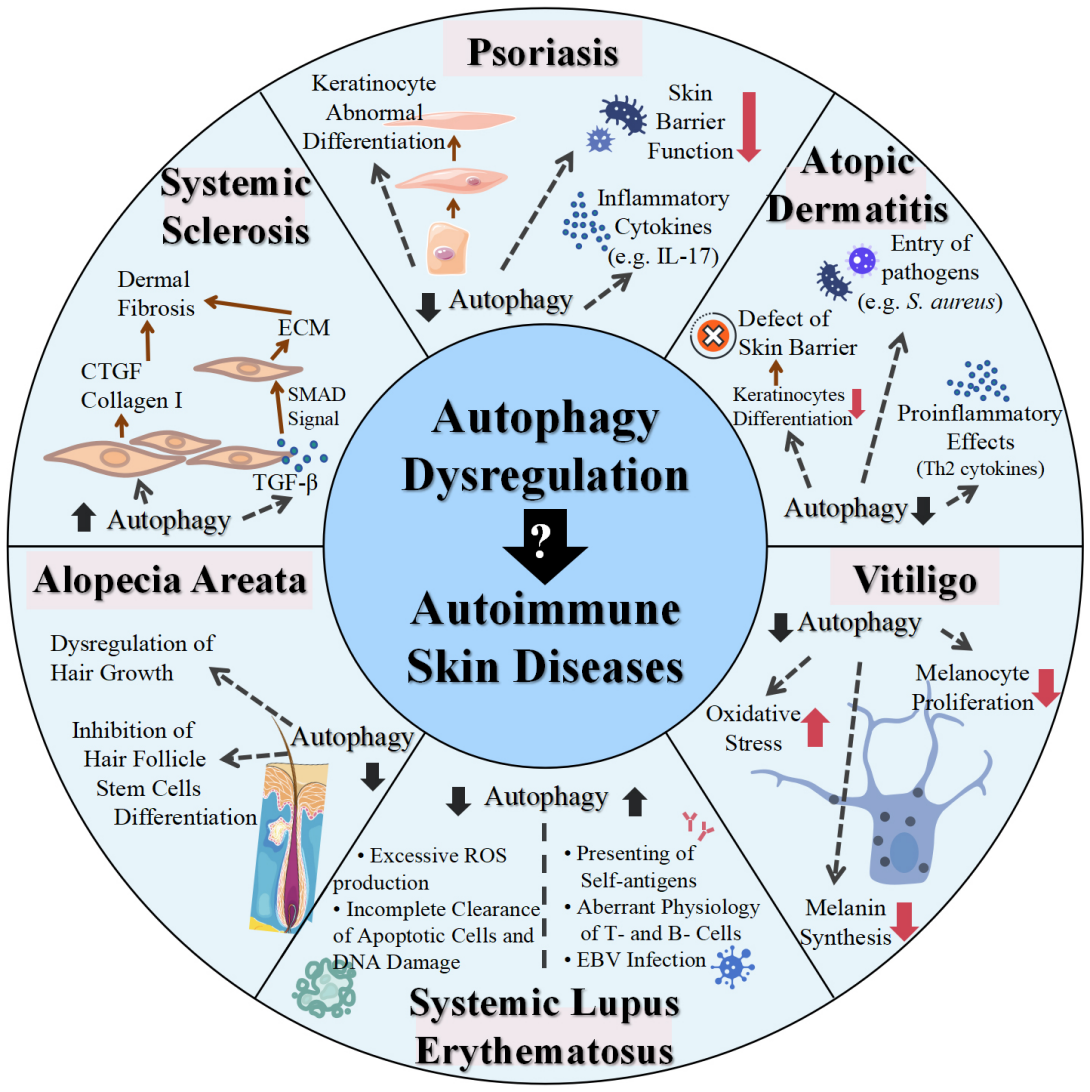
Role of dysregulated autophagy in various skin autoimmune diseases. IL, interleukin; *S. aureus*, *Staphylococcus aureus*; ROS, reactive oxygen species; EBV, Epstein–Barr virus; TGF, transforming growth factor; CTGF, connective tissue growth factor; ECM, extracellular matrix; SMAD, small mother against decapentaplegic.

Autophagy is an innate defense mechanism that maintains cellular equilibrium in response to different stress conditions by breaking down and recycling damaged or dysfunctional cellular components. Emerging evidence has established connections between autophagy and a multitude of immune functions, including defense against both intracellular and extracellular pathogens, modulation of the development and homeostasis of immune cells, and antigen processing and presentation, which positions autophagy as a mediator of autoimmunity. In the context of skin physiology, autophagy plays a pivotal role not only in regulating the metabolic and functional integrity of diverse skin cells but also in modulating skin inflammation and immune responses. Therefore, functional autophagy is closely associated with skin health and its proper functions, while the dysregulation of autophagy contributes to various skin disorders, particularly skin autoimmune disorders, as supported by polymorphisms in autophagy-related genes and altered expression levels of autophagic proteins observed in the forementioned skin autoimmune diseases (**Table 2**, **Figure 2**). In most cases, autophagy functions as a protective mechanism for skin autoimmune diseases. In the context of skin autoimmune diseases like psoriasis, AD, vitiligo, and AA, autophagy serves as a protective mechanism. The pathogenesis of those conditions is often associated with a reduced level of autophagy and disruption of its related pathways. This deficiency contributes to disease progression mainly by affecting skin barrier function, promoting inflammatory responses, disrupting the antioxidative defense mechanism, and impacting the homeostasis, development, and function of relevant cell types. In some circumstances, a moderate elevation of autophagy in these diseases tends to function as a compensatory mechanism. In contrast, in SLE and SSc, excessive autophagy has been observed, which may facilitate the progression of these diseases. Additionally, autophagy engages in complicated interactions with multiple types of cell death, which are also implicated in skin autoimmune disorders. For example, impaired mitophagy leads to pyroptosis and ferroptosis in certain skin diseases, such as psoriasis (144). Moreover, environmental factors, including UV exposure, smoking, and alcohol consumption, exert additional impacts on the diseases partially through the modulation of autophagy. Smoking has been reported to be associated with elevated risks of psoriasis and SLE (145, 146). The related mechanism could be attributed to oxidative stress, inducing abnormal mitochondrial functions and ER stress, which can be modulated through autophagy (146, 147). Excessive alcohol consumption is another risk factor for psoriasis, potentially related to the generation of ROS and its effects on both innate and adaptive immunity (148). Alcohol may not only induce ROS to activate autophagy but also increase p62 expression, which inhibits autophagy (149). To this end, the precise role of autophagy in the impact of alcohol consumption on psoriasis remains uncertain. Overall, autophagy plays a complex role in the pathogenesis of skin autoimmune diseases, with both excessive and deficient autophagy implicated in disease development. Therapies targeting autophagy-related proteins and signaling pathways have also been implicated in several autoimmune skin diseases. Glucocorticoids and rapamycin are both effective therapies for patients with SLE, which may relieve the symptoms of SLE by influencing autophagy (109). Notably, rapamycin, a pharmacological inducer of autophagy, exerts therapeutic effects mainly by binding to FKBP12 and inhibiting mTORC1 and its relevant pathways (150). The topical application of rapamycin improved imiquimod-induced psoriasis-like dermatitis, restored autophagy suppression, and reduced oxidative stress and the inflammatory response in the psoriatic mouse model (150, 151). Therefore, comprehensive research uncovering the functions of autophagy and its contributions to cell death-related and immune response-related machinery may provide a better understanding of the pathogenesis of skin autoimmune diseases. Both direct regulation of autophagy and synergistic use of autophagy modulators with conventional therapies may shed light on new therapeutic strategies for skin autoimmune disorders.

**Table 2:** Role of dysregulated autophagy in various skin autoimmune diseases.

Skin autoimmune diseases	Altered levels of autophagy-related genes and proteins	Alteration of autophagy	Role of dysregulated autophagy
**Psoriasis**	•Genes: *ATG16L1* and *AP1S3* (60, 70) •Proteins: ATG5, ATG7, Beclin1, LC3, and p62 (71–75)	**Reduced**	Abnormal differentiation and proliferation of keratinocytes (78, 79) Colonization of bacteria (76) Aggravation of inflammatory response (79, 80)
		**Induced**	Compensatory mechanism (78)
**Atopic dermatitis**	•Genes: *ATG4* and *ATG16L2* (85) •Proteins: ATG5, ATG7, LC3B, and p62 (75)	**Reduced**	Defect of skin barrier (86, 90) Proinflammatory effects (75, 86, 89) Entry of pathogens (91)
		**Induced**	Compensatory mechanism to re-establish cellular homeostasis (75)
**Vitiligo**	•Proteins: ATG4, ATG5, ATG7, ATG8, LC3, and Beclin1 (94, 95, 104)	**Reduced**	Suppression of melanogenesis (96) Impairment of melanocyte proliferation (100) Disruption of the antioxidant defense system (98, 99)
		**Induced**	A protector to counteract the progression of the disease (105)
**Systemic lupus erythematosus**	•Genes: *ATG5*, *ATG16L2*, *CDKN1B*, *DRAM1*, and *CLEC16A* (111, 112)	**Reduced**	An overload of damaged mitochondria and excessive ROS production (122) Incomplete clearance of apoptotic cells and DNA damage (125)
		**Induced**	Aberrant development, proliferation, and activation of T and B cells (114, 115) Processing and presentation of self-antigens (118) Epstein–Barr virus infection (130)
**Alopecia areata**	•Genes: *STX17, CLEC16A, BCL2L11*, and *ATG4B* (131–133) •Proteins: SQSTM1, ATG5, and LC3B (134)	**Reduced**	Dysregulated hair growth (135, 136) Inhibition of hair follicle stem cell differentiation (130)
		**Induced**	Protective mechanism in the early stages (128, 129)
**Systemic sclerosis**	•Proteins: LC3, Beclin1, ATG7, and p62 (108, 139)	**Induced**	Dermal fibrosis (141) Modulating the sensitivity of human fibroblasts to the profibrotic effects of TGF-β (139)

## Author contributions

YL: Visualization, Writing – original draft, Writing – review & editing. XW: Supervision, Writing – original draft, Writing – review & editing. YY: Writing – review & editing. YW: Writing – review & editing. LX: Supervision, Writing – review & editing. CZ: Conceptualization, Project administration, Supervision, Writing – review & editing.
